# The fat is in the lysosome: how *Mycobacterium tuberculosis* tricks macrophages into storing lipids

**DOI:** 10.1172/JCI168366

**Published:** 2023-03-15

**Authors:** Yoann Rombouts, Olivier Neyrolles

**Affiliations:** Institut de Pharmacologie et de Biologie Structurale (IPBS), Université de Toulouse, CNRS, UPS, Toulouse, France.

## Abstract

*Mycobacterium tuberculosis*, the causative agent of tuberculosis (TB), infects primarily macrophages, causing them to differentiate into lipid-laden foamy macrophages that are a primary source of tissue destruction in patients with TB. In this issue of the *JCI*, Bedard et al. demonstrate that 1-tuberculosinyladenosine, a virulence factor produced by *M. tuberculosis*, caused lysosomal dysfunction associated with lipid storage in the phagolysosome of macrophages in a manner that mimicked lysosomal storage diseases. This work sheds light on how *M. tuberculosis* manipulates host lipid metabolism for its survival and opens avenues toward host-directed therapy against TB.

## Foamy macrophages are a hallmark of tuberculous granulomas

*Mycobacterium tuberculosis*, the etiological agent of tuberculosis (TB), has plagued mankind for millennia and remains one of the deadliest pathogens today, as evidenced by the 10.6 million cases and 1.6 million deaths in 2021. *M. tuberculosis* is an intracellular pathogen that resides primarily within macrophages. *M. tuberculosis*–infected macrophages differentiate into lipid-laden foamy macrophages and aggregate together with other immune cells to form granulomas. Although granulomas generally serve as the physical and immunological barrier to bacterial growth and spreading (1, 2), *M. tuberculosis* has evolved to survive nutritional, hypoxic, and immunological constraints imposed by this environment. Furthermore, *M. tuberculosis*–infected foamy macrophages represent the primary source of caseous necrosis that causes tissue destruction and pulmonary cavitation in patients with TB (2–4). Accumulating evidence supports a model whereby *M. tuberculosis* induces reprogramming of host lipid metabolism to promote the accumulation of lipid droplets within the infected macrophages and uses these lipids as the primary carbon source for survival within the TB granuloma (4–10).

## 1-Tuberculosinyladenosine is a virulence factor of *M. tuberculosis*

Although macrophages are professional phagocytes equipped with a range of microbicidal weapons for killing intracellular microbes, *M. tuberculosis* has developed strategies to sidestep these defenses (1). In particular, the TB bacillus produces specific virulence factors that allow it to inhibit phagosome-lysosome fusion and autophagy, resist acidification of the phagolysosome, and disrupt the phagosome membrane to escape into the cytosol. In 2014, D. Branch Moody and collaborators performed a comparative lipidomic screen of *M. tuberculosis* and Calmette-Guérin bacillus (BCG), an attenuated *Mycobacterium bovis* strain used as a TB vaccine, and identified a *M. tuberculosis*–specific lipid they named 1-tubercolosinyladenosine (1-TbAd) (11). Since that initial discovery, 1-TbAd has been shown to be one of the most abundantly produced lipids in virulent *M. tuberculosis* strains, including clinical isolates (12, 13). Further studies have identified the 1-TbAd biosynthetic genes (*Rv3377c* and *Rv3378c*) and provided evidence that these genes appeared early in the evolution of the *M. tuberculosis* and were likely acquired by a horizontal gene transfer (11, 12, 14). Interestingly, a decade before the 1-TbAd discovery, genetic screening showed that *Rv3377c* and *Rv3378c* prevent phagolysosome acidification in macrophages and promote intracellular survival of *M. tuberculosis* (15). Consistent with these findings, Moody and colleagues demonstrated that 1-TbAd accumulates in acidic compartments, raising their pH, and causes lysosomes to swell in human macrophages (13). Importantly, these effects of 1-TbAd, especially phagolysosome swelling, could be recapitulated in human macrophages infected with wild-type *M. tuberculosis* but not with a 1-TbAd–deficient (*Rv3378c*-KO) mutant (13). Collectively, the results from these studies suggested that 1-TbAd is a *M. tuberculosis*–produced lipidic virulence factor that protects *M. tuberculosis* from macrophage-mediated intracellular killing. However, the link and exact mechanism governing these effects remain incompletely understood. In particular, when studying the mode of action of 1-TbAd, Moody and coworkers observed the formation of electron-dense inclusion bodies in swollen phagolysosomes of macrophages stimulated by 1-TbAd or infected by *M. tuberculosis*; however, they did not identify their nature.

## 1-TbAd causes lysosomal dysfunction and lysosomal lipid storage

**Figure 1:**
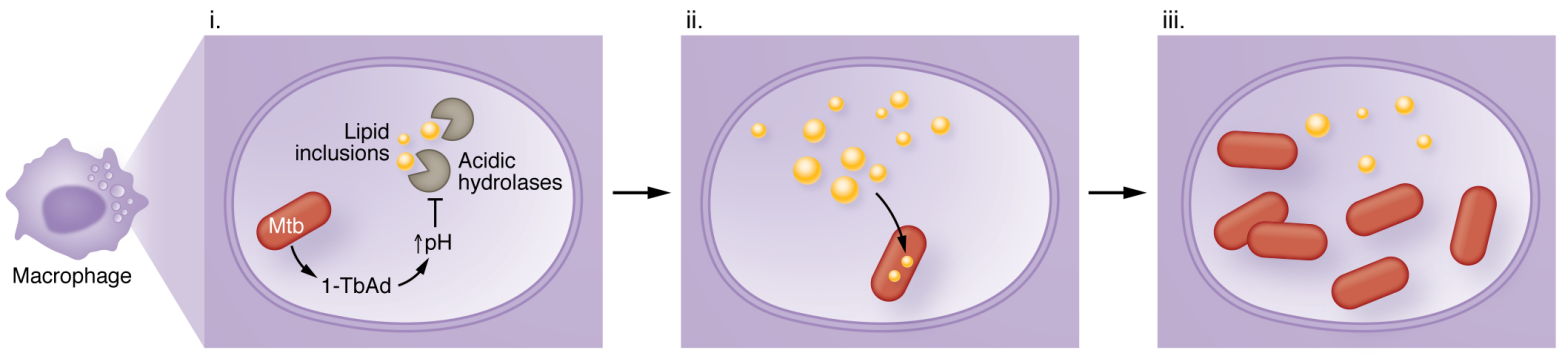
1-TbAd produced by *M*. *tuberculosis* induces lysosomal lipid storage. (i) *M. tuberculosis* (Mtb) produces 1-TbAd, which raises the vacuolar pH and inhibits acidic hydrolases, including lipases, inside the phagolysosome. (ii) Consequently, lipids, including cholesteryl esters and triglycerides, accumulate in the vacuole, mimicking lysosomal storage diseases. (iii) Under conditions of restricted lipid access, *M. tuberculosis* can use these lipids as a carbon source to promote its intracellular growth.

In this issue of the *JCI*, Bedard et al. (16) shine a light on these processes. By using correlative light and electron microscopy (CLEM), Bedard et al. demonstrate that the particular inclusions within swollen lysosomes of human macrophages were of a lipid nature, suggesting that 1-TbAd induced lipid storage in these cells (Figure 1). This finding was confirmed by immunofluorescence microscopy using BODIPY staining. A two-hour pulse of 1-TbAd was sufficient to trigger durable lipid accumulation in macrophages over several days. 1-TbAd–induced lipid accumulation in human macrophages was clearly related to lysosome remodeling, since this phenotype could be recapitulated with chloroquine, a classical lysosomotropic drug, but not with *N^6^*-tubercolosinyladenosine (*N^6^*-TbAd), a natural isomer of 1-TbAd with no lysosomotropism or antiacid properties (12, 13). To obtain a comprehensive picture of the lipids that accumulated in macrophages treated with 1-TbAd, Bedard et al. (16) performed lipidomic analysis and found that 1-TbAd increased the pools of cholesteryl esters and triacylglycerol, which are the main lipids that accumulate in *M. tuberculosis*–induced foamy macrophages (2–4). 1-TbAd also enhanced the amounts of monoalkyl-diacylglycerol, β-glucosylceramide, and lactosylceramide, which are lysosomal hydrolase substrates known to be stored in specific lysosomal storage disorders, including Wolman and Gaucher diseases.

On the basis of these results, Bedard et al. (16) hypothesized that by raising lysosomal pH, 1-TbAd inhibits the activity of intralysosomal acid hydrolases, resulting in the accumulation of lipids in this compartment. To address this hypothesis, the authors used enzyme-activated fluorogenic probes and showed that 1-TbAd strongly inhibited the acid-dependent glycosidase and protease activity within intact macrophages. Lysosomes play a fundamental role in the autophagic process by fusing with autophagosomes and degrading their contents. Consequently, the autophagic flux can be strongly disrupted as a consequence of any lysosomal dysfunction, as observed in lysosomal storage diseases or when using lysosomotropic drugs such as chloroquine. In agreement, Bedard et al. found that 1-TbAd, but not *N^6^*-TbAd, induced the accumulation of autophagosomes in macrophages, most likely by impairing autophagosome-lysosome fusion. Next, Bedard et al. asked whether direct effects of 1-TbAd on lysosomal lipid storage could also be observed in macrophages infected with 1-TbAd–producing *M. tuberculosis* strains. Electron microscopy (EM) analyses combined with immunogold staining for CD63, a lysosomal marker, confirmed, that infection with wild-type *M. tuberculosis*, but not with the *Rv3378c*-KO strain, which does not produce 1-TbAd, induced swelling of macrophage phagolysosomes. In addition, immunofluorescence microscopy revealed a substantial induction of lipid inclusions (indicated by Nile red staining) at day four after infection with wild-type and complemented bacteria, compared with the 1-TbAd–deficient mutant. Lipid inclusions in *M. tuberculosis*–infected macrophages were clearly localized to phagolysosomal (i.e., LAMP1^+^) compartments, as revealed by CLEM (16).

Since some lipid inclusions were observed in the phagolysosomes that contained bacteria, Bedard et al. then wondered whether *M. tuberculosis* could use these lipids, in particular cholesterol, as a carbon source to grow inside these cells. To answer this question, they measured intracellular *M. tuberculosis* growth in the presence of all-*trans*-retinoic acid (ATRA), which limits *M. tuberculosis* growth by restricting access to cholesterol (8), alone or in combination with 1-TbAd. Remarkably, treatment with 1-TbAd, but not *N^6^*-TbAd, abrogated ATRA-mediated restriction of *M. tuberculosis* growth in macrophages in a dose-dependent manner, suggesting that 1-TbAd–induced lipid storage in phagolysosomes provides nutrients to *M. tuberculosis* to support bacterial growth. Finally, Bedard et al. sought to assess whether the lysosomal failure of macrophages triggered by 1-TbAd could be reversed using pharmacological regulators of lysosomal activity. In particular, they focused on an agonist (C8) of transient receptor potential mucolipin channel 1 (TRPML1), a lysosomal Ca^2+^ release channel that prevents lysosomal dyshomeostasis (16–20). As hypothesized, treatment of 1-TbAd–stimulated macrophages with C8 decreased the size of swollen lysosomes by more than two-fold, dramatically reduced the number of lipid inclusions, and restored glycolipid catabolism (16).

## Conclusion and perspectives

Bedard et al. (16) demonstrate that 1-TbAd produced by *M. tuberculosis* caused lysosomal failure and accumulation of lipids in macrophages, mimicking lysosomal storage diseases. Furthermore, their data suggest that *M. tuberculosis* used lipids stored in lysosomes to support its growth inside macrophages, especially under conditions of limited lipid access. Finally, they show that 1-TbAd–induced, lysosome-dependent lipid accumulation could be counteracted by the use of a drug that restores lysosomal function.

These findings raise several questions and open several perspectives. First, 1-TbAd–induced lysosomal lipid storage could only be observed in human macrophages primed with both granulocyte-macrophage colony-stimulating factor (GM-CSF) and M-CSF (indicating M1 macrophages), but not, or only slightly, in M-CSF–polarized human macrophages (suggesting M2 macrophages) or mouse alveolar macrophages. In contrast, 1-TbAd swelled lysosomes in all these macrophage subtypes. The reason why 1-TbAd induces lipid accumulation specifically in M1 macrophages remains to be clarified. However, this apparent discrepancy could be linked to cell-specific differences in lipid handling and metabolism, since alveolar macrophages and M2 macrophages are committed to fatty acid oxidation, whereas M1 macrophages rely mainly on glycolysis for energy generation (21, 22). Second, foamy macrophages observed during *M. tuberculosis* infection have often been reported to be filled with lipid droplets, a phenotype not observed in this study (16). Thus, it is yet to be determined whether the unconventional, so-called “foamy” macrophages induced by 1-TbAd can be found in the host during TB infection. Nonetheless, the results of Bedard et al. (16) provide a possible explanation for some earlier observations, such as that intracellular *M. tuberculosis* can readily acquire lipids from macrophages, even when macrophages do not form lipid droplets (23). Finally, Bedard et al. (16) provide evidence that the use of specific lysosome-targeting drugs can reverse 1-TbAd–induced lysosomal dysfunction. Although future studies are needed to confirm these results in *M. tuberculosis*–infected macrophages and in vivo, this study provides initial evidence that lysosomes may be an attractive therapeutic target for the development of host-directed therapies against TB.

## Version 1 03/15/2023

Electronic publication
